# Recurrent Bacterial Vaginosis: A Case Report and Review of Management

**DOI:** 10.7759/cureus.37348

**Published:** 2023-04-09

**Authors:** Fady Safwat, Sandra Safwat, Natashah Daka, Nivetha Sivanathan, Milenko Lazarevic

**Affiliations:** 1 Medicine, Washington University of Health and Science, San Pedro, BLZ; 2 Medicine, Windsor University School of Medicine, Cayon, KNA; 3 Medicine, Richmond Gabriel University, Arnos Vale, VCT; 4 Internal Medicine, Swedish Hospital, Chicago, USA

**Keywords:** metronidazole resistance, human papillomavirus (hpv), recurrent bacterial vaginosis, bacterial vaginosis (bv), bacterial dysbiosis

## Abstract

Recurrent and refractory bacterial vaginosis is a potentially hazardous condition that affects the age-bearing population of women. We report the case of a 33-year-old patient presenting with recurrent bacterial vaginosis after attempting multiple regimens for the past three years. The patient had a history significant for ectopic pregnancy and multiple sexually transmitted diseases. Successfully managing this condition in the female population is crucial to prevent uncommon complications. Furthermore, introducing healthy vaginal microbiota can be the best course of action amongst patients with long-term recurrence of bacterial vaginosis.

## Introduction

Bacterial vaginosis (BV) is one of the most common gynecological conditions characterized by the disruption of vaginal acidity and microflora, resulting in the overgrowth of anaerobic bacteria in the lower genital tract. Most women with BV present with a complaint of malodorous vaginal discharge that worsens following sexual activity. Dysuria, dyspareunia, and vaginal pruritus are possible additional symptoms, but many affected women may be asymptomatic [1]. *Gardnerella vaginalis* is the most common causative agent, but *Prevotella* and *Mobiluncus* are also common anaerobic bacteria. About 30% of females in the United States between the ages of 14 and 49 are afflicted. However, percentages vary depending on the ethnic group and are most prevalent in non-White females (51% African Americans, 32% Mexican Americans) [1]. There is a global prevalence of 20-30% of BV infections among females of reproductive age, but several risk factors increase the possibility of contracting the disease [2]. These factors include multiple partners, use of antibiotics, smoking, and contraception via intrauterine devices or hormonal contraception [2]. Untreated BV increases the risk of sexually transmitted infections (STIs), including HIV and pregnancy complications. The likelihood of contracting gonorrhea or chlamydia after BV appears to increase by 1.9 and 1.8 times, respectively. The preceding risk factors can alter the normal flora, increasing the likelihood of acquiring BV [2]. Women of African American origin and those from low socioeconomic backgrounds had the highest prevalence rates.

The Centers for Disease Control and Prevention (CDC) has provided two primary diagnostic considerations for diagnosing BV. The first is the Nugent score, which calculates the relative concentration of lactobacilli in the vaginal lumen [3]. A score of 0-3 correlates to a *Lactobacillus*-predominant vaginal microbiota, 4-6 signifies the emergence of *G. vaginalis*, and 7-10, is consistent with an active BV. The other diagnostic consideration is the Amsel criteria which require three out of four clinical criteria to diagnose BV [3]. They include the following: clue cells (vaginal epithelium coating the bacterium) on microscopy, pH of >4.5, fishy odour of vaginal discharge before or after potassium hydroxide (KOH) addition (known as the whiff test), and the presence of a homogeneous, thin discharge [3]. The best treatment option for anaerobic bacteria has long been metronidazole and clindamycin for those with BV [4]. However, typically patients who initiate these drugs as monotherapy fail therapy after one month, and BV's recurrence rate can be between 50-80% after one year following treatment with either drug [5]. In vitro, antibiotic sensitivity testing on 50 strains of *G. vaginalis* to metronidazole and clindamycin showed that 68% of the isolates were resistant to metronidazole while 76% showed sensitivity [3-5]. Current clinical management for recurrent BV involves a combination of metronidazole and clindamycin, both potent disruptors of bacterial protein synthesis and alternating between oral and intravaginal administration.

## Case presentation

We present the case of a 33-year-old African American female who was evaluated in the clinic for a suspicious odour and thin vaginal discharge that was white. The patient was recently hospitalized for an ectopic pregnancy two months before the evaluation. The diagnosis was confirmed with a urine pregnancy test showing an elevated beta-human chorionic gonadotropin (b-HCG) level of 724.3 mIU/mL (normal range: <5 mIU/mL). After an extensive history was taken, the patient stated she had been experiencing these symptoms non-consecutively for the past three years despite taking multiple medications and trying home remedies. Current medications included a vaginal metronidazole cream and clindamycin 2% vaginal cream. Oral variations of these medications proved ineffective in reducing or eliminating the symptoms. The patient did not consume alcohol, smoke, or use drugs recreationally but admitted to infrequent condom use with one partner over the past year. Past medical history was significant for depression and recurrent migraines, managed successfully with escitalopram and Tylenol, respectively. Family history was non-contributory. Clinical examination at the visit revealed a well-appearing woman with no complaints of abnormal or heavy menstrual bleeding, dysuria, or tenderness in the lower abdomen. The patient was vaccinated against human papillomavirus (HPV) and discharged with oral metronidazole 750 mg for seven days and clindamycin 2% cream to be taken before bedtime. The patient was advised to follow up closely with her gynaecologist for further workup and to maintain safe sex and proper hygiene standards continuously.

## Discussion

Bacterial vaginosis is a relatively common and, in some cases, self-limiting condition. It may resolve spontaneously or recur if undetected and untreated, increasing the risk of sexually transmitted diseases STDs). Prolonged cases of BV have been associated with a predisposition to conditions like pelvic inflammatory disease, ectopic pregnancies, and induction of early labour if pregnant. In a case-control study by Zhang et al. (2021), a cohort of females with confirmed tubal pregnancies exhibited higher concentrations of *Gardnerella* over two years [6]. In this case, we can appreciate the development of ectopic pregnancy, which likely developed due to recurrent and refractory BV. This occurrence highlights the significance of prompt treatment for all symptomatic cases of BV. A study by Sobel et al. (2019) found that with 90 patients with recurrent BV, the reported mean scores were calculated to be pH 5.7, Nugent 8.6, and Amsel 3.9 [7]. A staggering majority of the patients were African American (90%). While more extensive studies on more diverse groups need to be conducted, recurrent BV could be more prevalent in African American women due to lower *Lactobacillus* colonization, as per the Nugent score. 

Recommended guidelines for the treatment of refractory BV include first targeting non-pharmaceutical measures, including smoking cessation, intrauterine device (IUD) removal, and instructions on safe sex practices [8]. This is typically followed up with either clindamycin or metronidazole, either orally or intravaginally, for seven days as an induction treatment. Breakthrough reoccurrences should be managed by switching to an alternative drug and changing the administrative technique [8]. Continued treatment failure should be treated with a trial of high-dose vaginal metronidazole of 750 mg for seven days and vaginal boric acid of 600 mg daily for seven days [8]. The persistence of BV after this warrants the addition of induction treatment along with a course of probiotics; in extreme cases, a vaginal microbiome transplant (VMT) should be considered [9]. Beyond this, there is no treatment directive apart from re-initiating the entire regimen. Apart from the VMT, our case of the 33-year-old female presenting with BV symptoms for the past three years and refractory to all other therapeutic guidelines is quite significant. It strongly raises concern about the worsening efficacy of the antibiotics used with anaerobic infections. It also raises the concern of tertiary prevention of patients with recurrent BV. A case series by Lev-Sagie et al. showed that four out of five patients who experience symptomatic and recurrent bacterial vaginosis achieve complete long-term remission between 5-21 months after being treated with VMT [10]. This was accomplished with no adverse effects noted. 

Pathophysiologically, BV results in an increased vaginal pH which sabotages the ideal environment for *Lactobacillus*, a native bacterium that can induce vaginal epithelial cell proliferation and d-lactic acid production [11]. This disruption can lead to STIs like *Chlamydia trachomatis* elemental bodies internalizing due to epithelium breakdown and an increased risk of cervical intraepithelial neoplasia through human papillomavirus (HPV) colonization [11]. In a study by Kovachev (2019) that included 32 women aged 38-55 years with pathology-established cervical cancer, 71.9% showed evidence of disruption to the vaginal microbiota [12]. Bacterial vaginosis accounted for 46.9% of the women who experienced dysbiosis [12]. These studies show an association between patients with recurrent BV and HPV infections. This shows the possible necessity and importance of HPV vaccination and safe sex practices.

## Conclusions

Recurrent BV has been shown to present with severe complications in females aged 16-44, from uncomplicated STDs to ectopic pregnancies, as seen in this case. The ineffectiveness of antibiotics in this case, coupled with the increasing severity of symptoms over the past three years, is very significant. This report aims to highlight the efficacy of therapeutic methods and prevention tactics to reduce the number of complications in a common infection. The best treatment for anaerobes has long been metronidazole and clindamycin in patients with BV. More randomized control studies are indicated by the lack of newer and effective therapies and regimens. In the absence of new, more effective antimicrobials to eradicate drug-resistant pathogenic vaginal microbiota and treatment advances in refractory and recurrent BV, clinicians should employ new strategies incorporating combination therapy, such as using combination antimicrobial regimens and alternative approaches such as probiotics and vaginal microbiome transplant. Until then, patients with recurrent BV may only benefit from a high-intensive drug regimen and prophylaxis against potential complications.
